# Point of care testing for high-sensitive troponin measurement: experience from a tertiary care hospital clinical laboratory

**DOI:** 10.1515/almed-2024-0058

**Published:** 2024-10-14

**Authors:** Sarra El Amrani, Bastien Tossens, Louisa Van Belle, Judit Gonda, Sherine Midoun, Christophe Beauloye, Damien Gruson

**Affiliations:** Faculty of Pharmacy UCLouvain, Brussels, Belgium; Department of Clinical Biochemistry, Cliniques Universitaires St-Lux, Brussels, Belgium and UCLouvain, Brussels, Belgium; North-Pest Central Hospital – Military Hospital, Budapest, Hungary; Division of Cardiology, 70492 Cliniques Universitaires Saint-Luc, Université Catholique de Louvain, Brussels, Belgium; Pole of Cardiovascular Research, Institut de Recherche Expérimentale et Clinique, Université Catholique de Louvain, Brussels, Belgium; Pôle de Recherche en Endocrinologie, 70492 Diabète et Nutrition, Institut de Recherche Expérimentale et Clinique, Cliniques Universitaires Saint-Luc and UCLouvain, Brussels, Belgium

**Keywords:** myocardial infarction, troponin, biomarker, point of care, efficiency, cost

To the Editor,

We are writing to share our insights and experiences with the evaluation of the Atellica VTLi^®^ (Siemens Healthineers) Point of Care Testing (POCT) system for high-sensitive troponin I (HsTnI) measurement at Cliniques Universitaires Saint-Luc (CUSL) in Brussels, Belgium. This system enables rapid HsTnI testing, delivering results within 8 min, which can significantly enhance clinical decision-making, particularly in acute coronary syndrome (ACS) scenarios where timely diagnosis and intervention are critical. In combination to clinical history, symptoms, vital signs and other physical findings, troponin measurement is a key element for the diagnosis of ACS [1]. In the latest ESC guidelines, it is clearly recommended to measure cardiac troponins with high-sensitive assays at the 0 h/1 h or 0 h/2 h intervals and to obtain results within 60 min of blood sampling [1]. POCT provides an alternative to tests traditionally conducted in central clinical laboratories, allowing testing close to the patient with a short turn-around time of analysis [2]. New generation of POCT testing for measurement of troponin with high sensitivity is now available and offer novel perspective for rapid patient triage [3]. The Atellica VTLi^®^ is a POCT device that can be performed on whole blood or plasma samples, and good clinical [4, 5] and analytical [6] performances have already been reported.

Our study aimed to evaluate the usability of the Atellica VTLi^®^ POCT system. Plasma samples were used for this evaluation. We conducted a survey involving eight healthcare professionals from various training backgrounds. The questionnaire was drawn up using the Scandinavian evaluation of laboratory equipment for POCT (SKUP) [7]. For each question (11 in total), the evaluator had to choose a score (satisfactory, intermediate, unsatisfactory). Obviously, the objective for user-friendliness is to achieve a satisfactory total score. The results are presented in the Table 1. The feedback was overwhelmingly positive. Users reported high levels of satisfaction with the device’s weight, test description, and sampling procedure. Notably, they indicated that no specific training was required, as the test was intuitive and straightforward. This aspect of user-friendliness is particularly important in emergency settings where the ease of use can significantly impact the efficiency of clinical workflows. The device size and the equipment required for test execution were rated as intermediate, further supporting its usability in diverse clinical settings. The “intermediate” scores highlight that while the POCT system is highly beneficial for urgent diagnostic purposes, its broader applications may require further training, adjustments in clinical protocols, or additional validation to optimize its use for risk prediction and ongoing patient monitoring. These mixed ratings emphasize the importance of tailored implementation strategies that address specific clinical needs and scenarios.

**Table 1:** Survey.

Criteria	Satisfactory (S)	Intermediary (I)	Unsatisfactory (U)	Results
Device weight	<5 kg	>5 kg × <10 kg	>10 kg	SSSSSSSI
Device size	<5 cm × 5 cm	>5 cm × 5 cm × <10 cm × 10 cm	>30 cm × 30 cm	SSSIIIII
Additional equipment needed to perform the analysis (device, cartridges, consumables)	No	1–2	>3	SSSSSSII
Description of the sample collection process	Yes	Incomplete	No	SSSSSSSS
Description of the test process	Yes	Incomplete	No	SSSSSSSS
Description of how the results are presented (and the units used are the same as those used for the routine testing)	Yes	Incomplete	No	SSSSSSII
Function and use of the test (in which cases it can be used: diagnosis/risk prediction/monitoring)	Yes	Incomplete	No	SSSSIIII
Presence of the necessary instructions for the user to perform the test correctly	Yes	Incomplete	No	SSSSSSSS
Training required to collect samples	No	Incomplete	Yes	SSSSSSSS
Training required to use the device	No	Basic training	Special training	SSSSSSII
Training necessary for the calibration and quality control of the device (presence of instructions to carry them out)	Yes	Incomplete	No	SSSIIIIU

**Figure 1:**
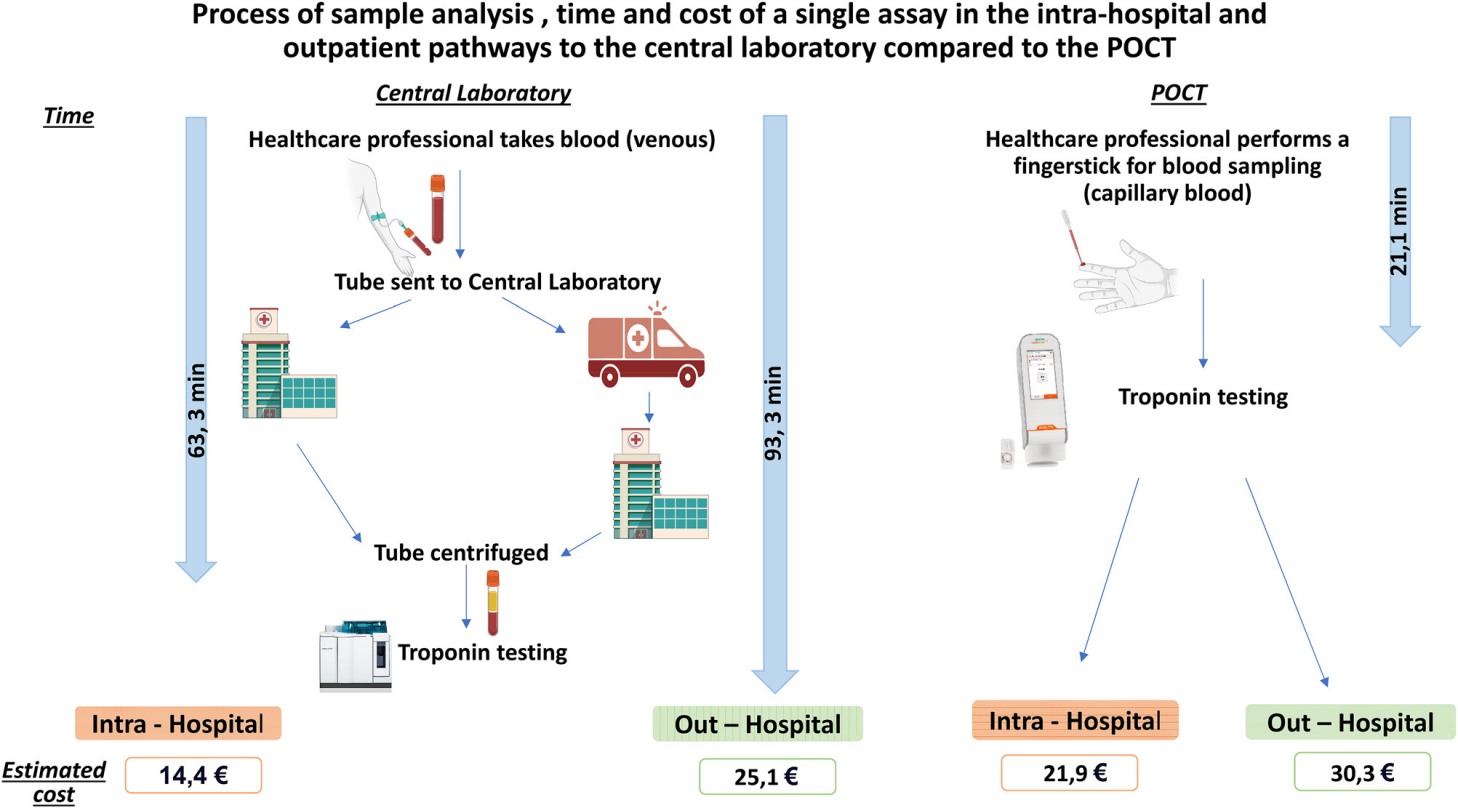
Process of sample analysis for POCT vs. Central Laboratory at Saint-Luc. Time and cost of a single assay in the intra-hospital and outpatient pathways to the central laboratory compared to the VTLi.

Another key component of our study was the comparison of the POCT pathway with the central laboratory pathway in terms of costs and turnaround time (TAT). The steps from patient presentation to result availability were meticulously recorded and analyzed. TAT from sample collection to result availability was recorded for both pathways, accounting for transportation in the central lab setting and on-site testing for POCT. The estimation of costs for point-of-care POCT using the Atellica VTLi^®^ system and central laboratory testing was performed based on a comprehensive analysis of various cost components involved in both pathways. The criteria for this cost estimation included direct and indirect costs associated with the testing process. For both POCT and central laboratory tests, common consumables such as alcohol swabs, gloves, adhesive bandages, syringes, needles, and tubes were included, with specific consumables for POCT including lancets and test cartridges. Equipment and instrumentation costs for POCT encompassed the Atellica VTLi^®^ instrument, computer software, quality control reagents, a refrigerator, and maintenance, while central laboratory costs included instruments, quality control reagents, and maintenance. Labor and staffing costs were calculated based on the time spent by healthcare professionals involved in sample collection, testing, and result processing, using hourly wages. Sample transportation costs for central laboratory testing included intra-hospital pneumatic tube transportation and ground transportation from primary care centers, which were eliminated in the POCT pathway. Infrastructure and overhead costs related to laboratory space and utilities were also considered. The POCT pathway demonstrated a substantial reduction in TAT. The median TAT for POCT was 21.1 min, significantly faster than the central laboratory pathway, which could take up to 93.3 min. Despite the faster TAT, the POCT pathway incurred slightly higher direct costs. The intra-hospital POCT pathway cost was 21.9 €, compared to 14.4 € for central laboratory testing. Similarly, the out-of-hospital POCT pathway cost was 30.3 €, compared to 25.1 € for central laboratory testing. Summary of the results is presented in the Figure 1.

While the direct costs of POCT are higher, the overall economic benefits may justify the investment. The rapid turnaround time facilitated by POCT can lead to several indirect cost savings. For instance, faster diagnosis and treatment can reduce the length of hospital stays, optimize the use of healthcare resources, and improve patient throughput. Previous studies have shown that faster troponin-based algorithms are cost-effective compared to standard management, with significant reductions in costs and length of stay per patient [8]. Moreover, the environmental impact of POCT should not be overlooked. Our current outpatient pathway involves sample ground transportation by car to the central laboratory. In contrast, POCT provides on-site results, reducing the need for transportation and its associated environmental footprint. This aspect contributes to more sustainable healthcare practices, aligning with broader goals of environmental responsibility.

In conclusion, our preliminary experience with the Atellica VTLi^®^ POCT system for HsTnI measurement at a tertiary care hospital underscores its potential to enhance clinical decision-making through rapid turnaround times. In ACS management, where “time is muscle,” the ability to quickly obtain and act on HsTnI results can significantly impact patient outcomes. The system’s user-friendly design and positive user feedback highlight its usability in diverse clinical settings. Although the direct costs of POCT are higher, the overall economic benefits, including faster patient triage and resource optimization, support its implementation. Further comprehensive studies are needed to fully assess the economic impact and validate these findings.
